# Supplementary figures and images for: Epigenomic Alterations of the Human CYP11B Gene in Adrenal Zonation
Source: Int J Mol Sci. 2024 Nov 7;25(22):11956. doi: 10.3390/ijms252211956 (PMC11593487; doi:10.3390/ijms252211956)

*CYP11B1*

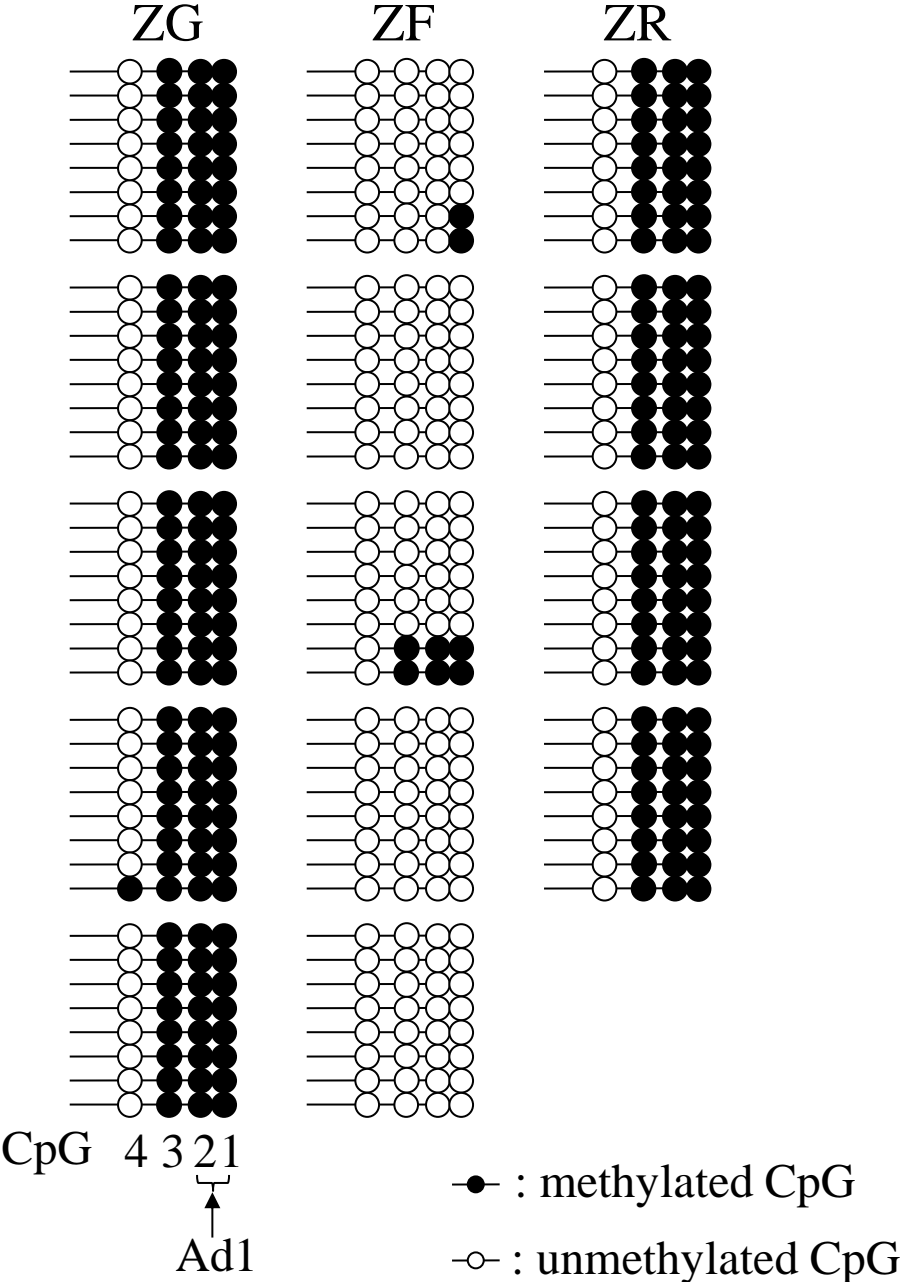

Supplement: Supplementary file 1 [file ijms-25-11956-s001.zip › ijms-3181240-supplementary.pdf]
